# Erratum

**DOI:** 10.1111/cas.14628

**Published:** 2020-09-03

In an article^1^ titled “Five‐day regimen of azacitidine for lower‐risk myelodysplastic syndromes (refractory anemia or refractory anemia with ringed sideroblasts): A prospective single‐arm phase 2 trial” by Yasuyoshi Morita, Yasuhiro Maeda, Terufumi Yamaguchi, Fumiaki Urase, Shuhei Kawata, Hitoshi Hanamoto, Kazuo Tsubaki, Jun Ishikawa, Hirohiko Shibayama, Itaru Matsumura, and Mitsuhiro Matsuda, Table 3 is to be corrected.

The wrong placement of the columns under “IPSS” and “IPSS‐R” resulted in two subgroups of low risk in IPSS and an inconsistent number of total patients between these two risk groups, a number which should be the same. Table 3 should be as shown:

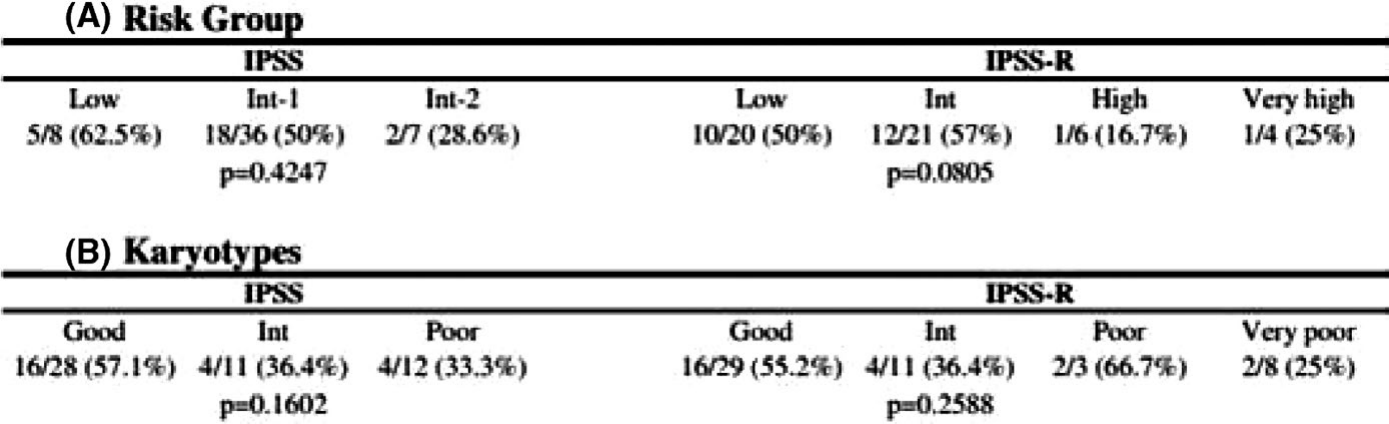

The therapeutic response was evaluated by International Working Group 2006 response criteria. (A) According to risk group and (B) according to karyotype in IPSS and IPSS‐R. Dichotomous variables were compared between different groups using the Fisher's exact test. Int, intermediate.

The Publisher apologizes for the errors.
